# Properties of Cold Sprayed Titanium and Titanium Alloy Coatings after Laser Surface Treatment

**DOI:** 10.3390/ma15249014

**Published:** 2022-12-16

**Authors:** Rafał Zybała, Bartosz Bucholc, Kamil Kaszyca, Krystian Kowiorski, Dominika Soboń, Wojciech Żórawski, Dorota Moszczyńska, Rafał Molak, Zbigniew Pakieła

**Affiliations:** 1 Faculty of Materials Science and Engineering, Warsaw University of Technology, 141 Woloska St., 02-507 Warsaw, Poland; 2 Łukasiewicz Research Network—Institute of Microelectronics and Photonics, 32/46 Al. Lotnikow, 02-668 Warsaw, Poland; 3 Institute of Fundamental Technological Research of the Polish Academy of Sciences, 5B Pawinskiego St., 02-106 Warsaw, Poland; 4 Faculty of Mechatronics and Mechanical Engineering, Kielce University of Technology, 7 Al. Tysiaclecia Panstwa Polskiego, 25-314 Kielce, Poland

**Keywords:** cold spray, laser surface treatment, titanium coating, Ti6Al4V, residual stresses

## Abstract

Additive manufacturing (AM) has seen remarkable development in recent years due to relatively high efficiency of the process. Cold spraying (CS) is a particular method of AM, in which titanium and titanium alloy powders are used. CS is a very competitive technology enabling the deposition of coatings, repairing machine parts, and manufacturing new components. For specific applications, the surface of cold-sprayed materials may require further processing. This paper reports an attempt to employ laser surface treatment (LST) of cold-sprayed coatings on an aluminium alloy substrate. The influence of laser beam interaction time on the coatings’ properties was analysed. The microstructure was investigated and observed employing scanning electron microscopy (SEM). To evaluate residual stress after CS and LST, the sin^2^ψ technique was used. Investigations were also performed on Vickers hardness, contact angle, and surface roughness. Significant changes in the surface morphology of the coatings and elevated residual stress levels dependent on the laser beam interaction time were observed. Increased Vickers hardness was recorded for titanium alloy Ti6Al4V. LST also led to increased surface hydrophilicity of the modified materials Ti and Ti6Al4V.

## 1. Introduction

In recent years, additive manufacturing (AM) is indisputably one of the most intensively developing fields of materials and production engineering. Amongst its inherent advantages that distinguish AM from other conventional techniques such as machining or casting, one should mention the reduction of material waste, increased production process flexibility, and the possibility of manufacturing parts with complex geometries [1,2,3,4,5]. The main research directions in the field of AM focus on microstructure and materials’ properties optimization, development of new, more advanced techniques, improvement of the production processes efficiency, and applying AM for new materials [2,3,6,7,8]. It should be emphasised that, in accordance with the classification proposed by Kumar and Kar [9], the general classification of additive manufacturing methods consists of two main categories: solid state and fusion based. To fabricate a part with the solid-state-based methods, the powder particles of material are bonded together without transitioning to a liquid state. In this case, other mechanisms such as pressure, friction or high powder particle velocities are employed. On the other hand, fusion-based techniques involve bonding powder particles together, e.g., by melting or sintering them with a laser beam or through a local melting of the wire of the material from which the component is being manufactured. The most commonly used additive techniques for metal components are selective laser melting (SLM), electron beam melting (EBM), directed energy deposition (DED), binder jetting (BJ), and cold spraying (CS). The latter differs significantly from the others in the mechanism of bonding material particles together. Other methods involve sintering or melting, while in CS, the particles are accelerated to high velocities (above critical) and then undergo localized severe plastic deformation. According to Grujicic et al. [10], the bonding mechanism is due to the adiabatic shear instability that occurs during impact, and it is a result of the high strain rate deformation process. Therefore, in the case of cold spray additive manufacturing (CSAM), bulk components or coatings are produced with no liquid phase involved. As opposed to the high-temperature deposition processes, this helps to avoid problems related to phenomena such as unfavourable phase transformation, oxidation, and residual thermal stresses [11,12,13,14]. It is a commonly used technique in automotive, aerospace, marine, medical, chemical and many other industries [11]. Amongst the most often cold sprayed materials are Al and Al alloys [11,14], Ni [15], Cu [16], steels [17], and Ti and Ti alloys [18,19,20].

Titanium possesses a very advantageous combination of properties such as high specific strength, good erosion and corrosion resistance, toughness, biocompatibility and biotolerance [21,22,23]. Due to its physical properties, it can also be applied as diffusion barriers preventing undesirable mixing of components in composites [24]. These features and the possibility of application in various AM techniques have induced the utilization of Ti and Ti-based alloys in a wide range of industries. Depending on the crystallographic structure, three main types of titanium alloys can be distinguished: α, β, and biphasic α-β. They exhibit different properties, e.g., α alloys have the highest strength and oxidation resistance at elevated temperature, creep resistance, and they are also the most easily weldable. β alloys show the best heat treatment response and much better hardenability than the others. Biphasic α-β alloys have superior mechanical properties, and their heat treatment is more effective than in the case of α alloys [25]. The last group includes over 70% of commercially used titanium alloys, including Ti6Al4V (Ti64) [21]. Ti64 is very popular in biomedical applications, especially for implants and prostheses. In addition, it is used to produce structural elements in the aviation, automotive, and maritime industries. For specific applications, parts or coatings produced with AM methods (especially cold-sprayed) may require further processing that results in optimal, desired surface properties. Such processing may be carried out by means of laser surface treatment (LST).

Laser surface treatment (LST, also laser surface engineering) was applied in the past to improve the properties of titanium and titanium alloys such as wear resistance, corrosion, and high-temperature oxidation resistance [26,27]. This was achieved by modifying the microstructure through melting of the surface followed by rapid cooling. Additionally, there were also attempts to combine melting of the surface layer with simultaneous interference in its chemical composition (laser surface alloying), e.g., by co-deposition of alloying elements on the surface during lasing [28]. The influence of the laser treatment (consisting in surface melting) parameters on the mechanical and electrochemical properties of Ti64 surface, as well as its microstructure, were described by Mann and Majumdar in their publication [21]. They noticed that surface melting with the use of a laser led to the formation of martensitic (α′) phase precipitates in the form of needles, which is characterized by a hexagonal close-packed crystal structure and a lattice parameter similar to the lattice parameter of α-phase. However, it shows greater hardness and inferior plasticity. According to the aforementioned investigations, laser treatment of the Ti64 alloy surface effectively improved the microhardness of this alloy compared to its as-received state—it increased from 280 VHN to 550 VHN. In addition to the applications mentioned above, LST is also one of the methods that could be used, for example, for surface cladding [29]. In addition to laser-based post-processing, techniques such as friction stir processing (FSP) can also be employed to enhance the performance of cold-sprayed coatings. This state-of-the-art approach, based on the example of CS 316L steel, was presented by Ralls et al. in their recent paper [30]. FSP is a technique that uses frictional heat generated between the active workpiece and the processing material, which leads to a dynamic recrystallization. This results in a microstructural refinement, which improves both mechanical and tribological properties. A considerable advantage of post-processing using the FSP technique is its high energy efficiency compared to other techniques.

Over past years, there has been an increased interest in LST of cold-sprayed materials. Khun et al. investigated the effects of Nd:YAG laser power on the tribological properties of a 3 mm thick Ti64 coating [31]. According to their research, LST significantly influenced wear resistance. Moreover, the higher the laser power applied, the higher hardness of the coating was measured. That was attributed to more rapid cooling, stemming from a greater temperature gradient between the coating and room temperature. The effect of laser melting of cold sprayed materials was investigated by Astarita et al. [32]. In their work, they mainly focused on the microstructure analysis of 5 mm thick titanium coating that was detached from the substrate and then laser treated. They noticed that in the base material zone and the heat-affected zone, the equiaxial grains of α phase were present, while in the melted zone, acicular grains of martensite α’ could be found. When it comes to the mechanical properties, the laser-melted zones exhibited higher Vickers microhardness. In addition to those researchers mentioned, Marrocco [33], Carlone [34], and Rubino [35] also studied the impact of the laser post-treatment of cold-sprayed titanium on steel or aluminium alloy substrates. They investigated the influence on, inter alia, corrosion resistance, microhardness, and microstructure of the obtained layers. Carlone’s work also presented a numerical model of laser processing, the results of which were consistent with the experimental results. However, none of this work attempted to analyse the level of residual stress state induced by the performed laser treatment.

The improvement of mechanical properties by the means of LST was also achieved by Sobon [36]. She deposited Ni20Cr on Al7075 substrate using the CS method and subsequently modified it with a 4 kW CO_2_ laser. The laser melting of a coating led to lowered porosity level, increased microhardness, and higher Young’s modulus. Furthermore, the microstructure evaluation revealed the change from lamellar, after CS, to columnar, resulting from the applied laser modification.

Since the cold spray process alone offers relatively limited control over the surface properties of additively manufactured components, coatings, or repaired parts, we decided to investigate the influence of laser processing on, inter alia, the phase composition, residual stress state, hardness, and surface condition of commonly used materials such as titanium and titanium alloy—TI6Al4V. A thorough understanding of the relationship between the surface properties of cold-sprayed materials and surface laser treatment parameters will allow a widespread use in industry and, thus, increase the applicability of cold-spray technology.

## 2. Materials and Methods

### 2.1. Materials and Sample Preparation

**Figure 1:**
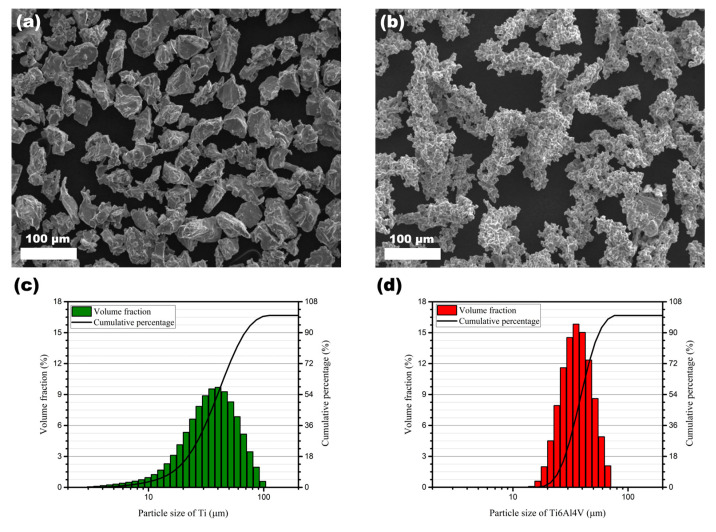
SEM images showing powders’ morphologies: (**a**) angular Ti, (**b**) spongy Ti64, and particle size distributions of (**c**) Ti and (**d**) Ti64.

Commercially available powders of titanium (99 wt% Ti) and Ti6Al4V (Ti64) were used for deposition of the cold-sprayed coatings, both manufactured by Kamb Import-Export. Scanning electron microscopy (SEM) images of morphologies and particle size distributions are depicted in Figure 1a,b and Figure 1c,d, respectively. As can be seen in Figure 1a,b, the powders of the two materials differ fundamentally. The Ti powder is angular, whereas Ti64 has a spongy structure.

Figure 1c,d show the powder particle size distributions of both titanium and titanium alloy—Ti6Al4V. The green and red columns refer to the volume fraction (left vertical axis), i.e., the percentage of powder particles of a certain size (horizontal axis). The cumulative percentage represents the sum of the specific powder particle fractions. Illustrated by a solid line, it refers to the right vertical axis of the graphs. These values were obtained using laser diffraction particle size analyser HELOS H2398 (Sympatec GmbH, Clausthal-Zellerfeld, Germany). Based on the distributions, Ti powder has a higher fraction of smaller particles, less than 20 µm. Both materials were cold sprayed on a 5 mm thick aluminium alloy (Al7075) substrate. Before the deposition process, the substrate’s surface was sandblasted with corundum grits to improve the adhesion of the coating. The surface roughness parameters of the substrate after treatment were as follows: Ra = 7.12 μm, Rz = 41.92 μm, Rq = 8.99 μm.

**Figure 2:**
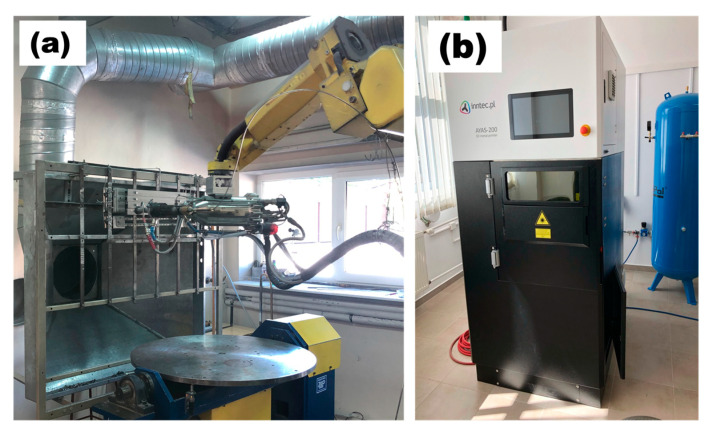
(**a**) Cold spray equipment, (**b**) 3D metal printer AYAS-200 used for laser surface treatment.

Both coatings (Ti and Ti64) were deposited by means of the cold spray (CS) method. For this purpose, Impact Innovations 5/8 Cold Gas Spray System equipped with the Fanuc M-20iA robot was used (Figure 2). As the propellant gas, a mixture of helium and nitrogen in a ratio of 1:1 at the pressure of 4 MPa was utilized. The supplementary process parameters are given in Table 1. The thickness of deposited Ti and Ti64 coatings were 923 ± 27 and 456 ± 26 µm, respectively (based on SEM image analysis).

**Figure 3:**
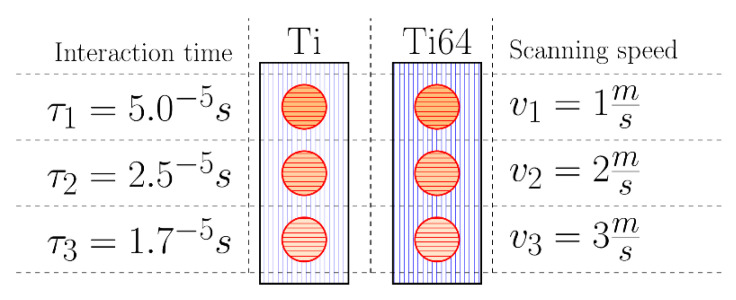
Samples layout during laser treatment process.

The laser integrated with a 3D metal printer AYAS-200 (INNTEC, Gdansk, Poland) was used for coatings’ laser treatment. It is a 200 W single-mode fiber laser with power regulation. The laser wavelength is 1070 ± 15 nm, the diameter of the laser beam, *d*, focused spot 50 µm, and the maximum scanning speed, *v_max_*, is 10 ms^−1^. After preliminary trials, all laser treatments of CS coatings were set to be performed with laser power 150 W, but with different scanning speeds ranging from 1 to 3 ms^−1^, and, therefore, different interaction times, *τ_i_*, of the laser beam with the material surface (Table 2). The interaction time, *τ_i_*, was determined as the ratio of the laser beam diameter, *d*, and the scanning speed, *v_i_*. Figure 3 presents the layout of samples during laser treatment. The labels are constructed as follow [ID−v], where ID can be either Ti or Ti64, and $v_{i}$ represents the scanning speed. The movement direction of the laser beam during LST (red lines) was perpendicular to that of the cold spray nozzle during the deposition process (purple lines).

**Figure 4:**
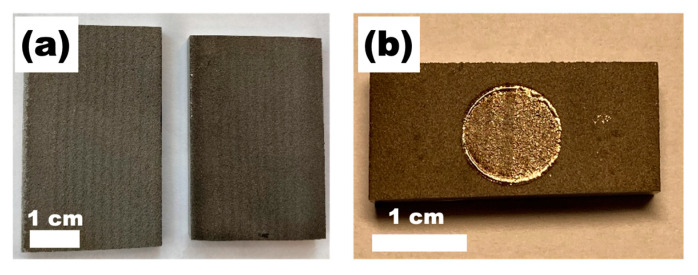
(**a**) Cold-sprayed coatings (Ti on the left and Ti64 on the right-hand side), (**b**) One of the laser surface treated areas.

Samples for LST were put in the chamber of the 3D printer on the steel platform heated up to 200 °C. The laser-treated areas were circles with a diameter of 11 mm. The as-deposited materials and the sample after LST are shown in Figure 4.

The wetting angle, surface roughness measurements and XRD analyses were performed on the laser-treated surface as such. For hardness investigations and selected SEM observations, the metallographic cross sections were prepared by cutting with a diamond cut-off wheel. Following cutting, the samples were embedded in the epoxy resin and then ground with progressively finer grades of SiC grit paper (800, 1200, 2400, 4000) and finally polished with a 3 and 1 µm diamond polish agent.

### 2.2. Microstructural and Phase Composition Investigation

To investigate the phase composition of base and laser-modified materials, X-ray diffraction (XRD) technique was utilized. Both qualitative and quantitative analyses were performed. The whole powder pattern fitting method (WPPF) was used. The measurements were conducted with the diffractometer Smartlab 3 kW (Rigaku, Tokyo, Japan), equipped with Cu Kα (1.5406 Å) radiation source operated at 40 kV and 30 mA, on the as-lased surface without prior removal of surface roughness. A *2θ* diffraction angle was 30–140°, and the scanning rate was 2°/min. The patterns used for the phase analysis were sourced from the International Centre for Diffraction Data database PDF-4 + 2021. The materials’ microstructures were analysed with the use of scanning electron microscopy (SEM) (Phenom ProX, Thermo Fisher Scientific, Waltham, MA, USA).

### 2.3. Residual Stress Measurement

For residual stress assessment, the sin^2^*ψ* method was used (where *ψ* is an angle between the normal of the sample and the normal of the diffracting plane). Measurements were made using Smartlab 3 kW diffractometer (Rigaku, Tokyo, Japan) equipped with 0D detector and Cu K*α* radiation in the parallel beam geometry (PB). The step size Δsin^2^*ψ* was 0.125. The diffraction peak corresponding to lattice plane (114) of the hexagonal close-packed crystal structure of α-Ti, as well-separated, high-indexed planes lying at c.a. 115° (2*θ*), exhibiting relatively high intensity, was chosen. For the stress determination, XRD measurements were made at 5 different *ψ* tilts (*ψ* = −45.0°; −37.8°; −30.0°; −20.7°; 0.0°). With the use of the PDXL2 software by Rigaku, the evaluated inter-planar spacing values, *d*, were plotted against the values of sin^2^*ψ* function. The gradient, *m*, of the resultant curve was determined. A plane stress state was assumed to be present in the investigated coatings. For both materials, Young’s modulus, *E*, and Poisson’s ratio, *υ*, were taken as 115 GPa and 0.34, respectively. Based on the assumptions made, taking the stated elastic properties of the materials, the residual stresses, *σ*, were calculated according to the following relation: *σ* = (*E*/(1 + *υ*)) *m*.

### 2.4. Wetting Angle, Microhardness and Surface Roughness Investigations

FM40 EasyDrop (Krüss GmbH, Hamburg, Germany) and drop shape analysis (DSA1.0 software) methods were utilized for wetting angle studies. Contact angles were determined as an average value of 5 measurements conducted on pictures of a deionized water drop taken 2 s after dropping.

The microhardness of the samples (before and after laser treatment) was determined using DuraScan 20 (Struers, Salzburg, Austria) equipped with the pyramidal diamond indenter. The Vickers hardness of the samples was determined as an average value of 10 indentations made on the prepared surface of cross-sections under a normal load of 0.1 kg (HV01) and loading time of 10 s. Standard error was determined.

The surface roughness investigations were carried out with the use of a Dektak 150 nanoprofilometer (Veeco, New York, NY, USA) on a measuring length of 6 mm. Roughness measurements were carried out in the direction perpendicular and parallel to the direction of the cold spray nozzle during the deposition process. The average Ra parameter (defined as an arithmetic average of profile height deviations from the mean [37]) was determined for every sample in both directions.

## 3. Results

### 3.1. X-ray Phase Analysis

**Figure 5:**
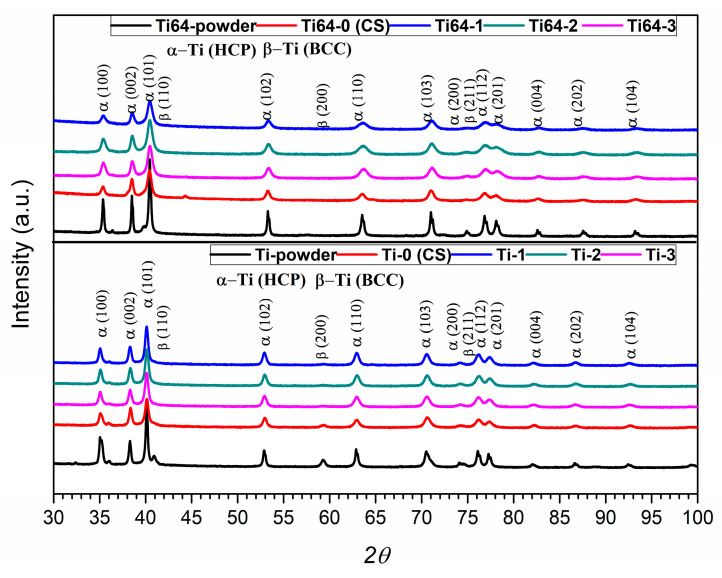
Set of diffraction patterns for Ti and Ti64 material for powders, cold-sprayed samples, and laser-treated coatings.

Figure 5 depicts a set of diffractograms for pure Ti samples. Based on the carried-out investigations for the feedstock powders, both were found to contain two phases. Based on the WPPF analysis method used, the α phase with a hexagonal close-packed crystal structure (HCP) is significantly dominant in both cases, while the body-centred cubic structure β phase (BCC) shows a much lower volume percent. However, it should be noted that the identified phases differ slightly in lattice parameters, which is naturally a consequence of the lattice distortion caused by the presence of alloying atoms. Diffractogram analysis showed that, for the as-deposited coatings materials as well as for the laser surface treated, virtually only the close-packed hexagonal phase is present, both for Ti and titanium alloy. Very low reflex intensity at 2*θ* = 41° and 59° and 75° positions, with regard to the α phase reflexes, indeed indicate the presence of a second phase, but the quantitative analysis performed reveals that its percentage is close to the measurement error of the technique used.

### 3.2. Microstructure and Morphology Analysis

**Figure 6:**
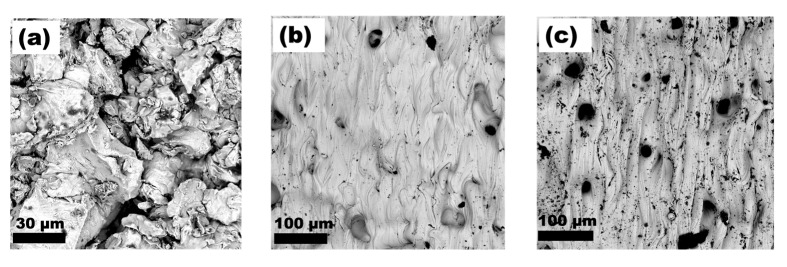
SEM images of surface morphologies (**a**) Ti-0, (**b**) Ti-3, (**c**) Ti-2.

Figure 6a shows the top view of the morphology of the cold-sprayed titanium coating. The voids between the powder particles are visible. To a great extent, the powder particles on the top coating’s layer retained their original angular shape they had in their initial state. In contrast, the morphology of the titanium layer subjected to laser surface modification at speeds of 3 and 2 ms^−1^ is shown in Figure 6b,c, respectively. For the latter, one can see many open pores with a diameter of several tens of microns. Furthermore, there are distinct traces of a laser beam path about 50 microns wide.

**Figure 7:**
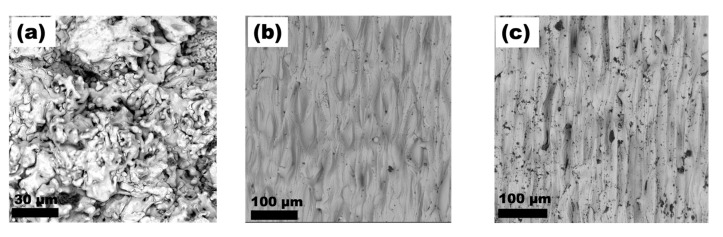
SEM images of surface morphologies (**a**) Ti64-0, (**b**) Ti64-3, (**c**) Ti64-2.

The morphology of the cold-sprayed titanium alloy coating can be seen in Figure 7a. As in the case of titanium with no alloying elements, some of the powder particles constituting the top layer of the coating retained their initial morphology—described as spongy or coral-like. Similar to Ti, numerous voids are also visible for Ti64. In Figure 7b, an area of the laser-modified Ti64 surface can be seen at the highest beam scanning speed of 3 ms^−1^. In this case, as for all laser-modified Ti64 surfaces, the presence of open pores on the surface was not so pronounced as for Ti.

**Figure 8:**
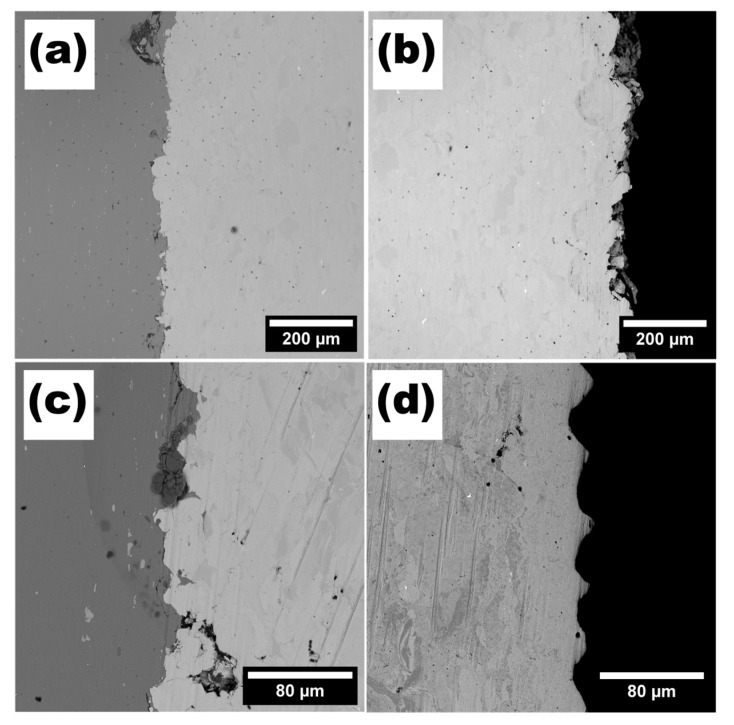
Cross-sections of (**a**) Ti-0 aluminium–titanium junction, (**b**) Ti-0 titanium layer, (**c**) Ti-1 aluminium–titanium junction, (**d**) Ti-1 titanium laser-modified layer.

Figure 8a,b present the morphology of CS-deposited Ti layers before LST. The titanium layers have high density and exhibit excellent adhesion to the base material. Figure 8c shows junction between base material and the laser-treated Ti layer. The laser-treated layer surface (Figure 8d) shows visible laser traces.

**Figure 9:**
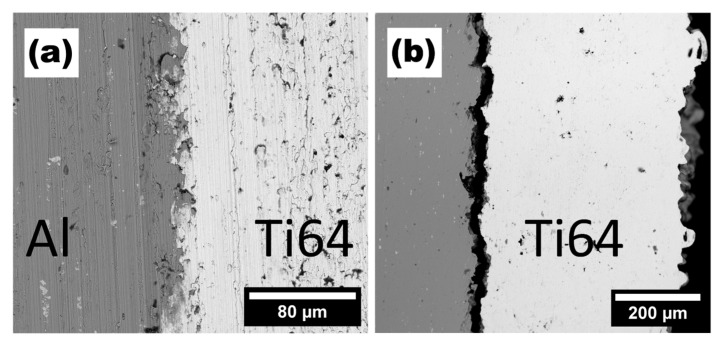
(**a**) Ti64 layer before laser treatment (**b**) Ti64-3 sample after laser treatment.

The CS deposited Ti64 layer is presented in Figure 9a. The Ti64-0 layer shows good adhesion between base material. There is a visible porosity inside the Ti64 layer. All Ti64 layer-base joins cracked after laser treatment process—there is a visible gap between layers in all modified layers. Due to cracking within all the Ti64 laser modified layers [*v* = 1, 2, 3 ms^−1^], this branch will not be described in detail.

### 3.3. Residual Stresses

**Figure 10:**
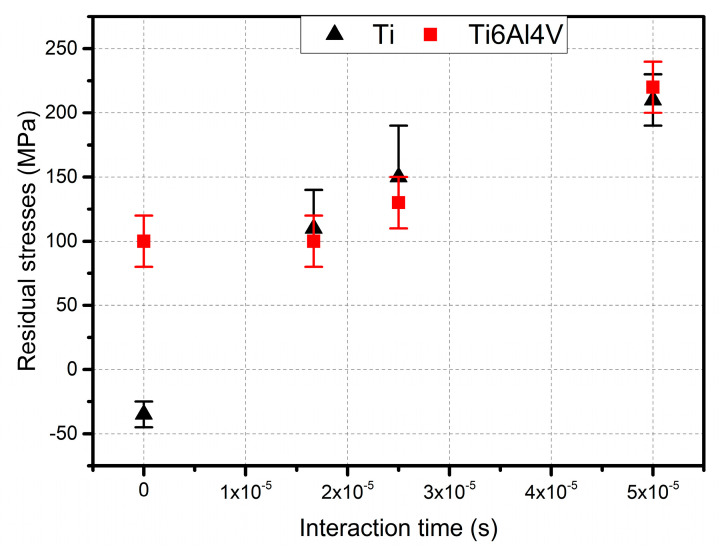
Residual stress in Ti and Ti64 samples before and after the SLT, determined with the sin^2^*ψ* method.

The results of the residual stresses determined using the sin^2^ψ method for the samples after the CS deposition process, and the laser-modified coatings are shown in Figure 10. The visible error bars of measurements represent a variance (σ^2^) and are largely due to the specificity of the diffraction measurement method. For the commercially pure Ti sample after the CS deposition process, a compressive stress of −35 ± 10 MPa was observed. As a result of the laser treatment, an increase in stress values correlated with the increasing interaction time, τ_i_, of the laser beam with the coating surface was noted. For the shortest interaction time (thus, the highest beam scanning speed), an increase in stress value from −35 ± 10 up to 110 ± 10 MPa was evident. These residual stresses increased with decreasing laser beam speed, up to over 200 MPa for the speed of 1 ms^−1^. The laser-treated Ti64 alloy showed residual stress values similar to pure Ti. As for Ti, they increased with increasing beam interaction time, taking values close to those measured for pure Ti. In contrast, a different situation was observed for the as-deposited Ti64 sample. In this case, the stress values were significantly higher than for Ti (about 100 MPa) and were different tensile. However, it should be noted that the thicknesses of the Ti and Ti64 coatings were different, which can affect the value of the residual stresses.

### 3.4. Vickers Hardness

**Figure 11:**
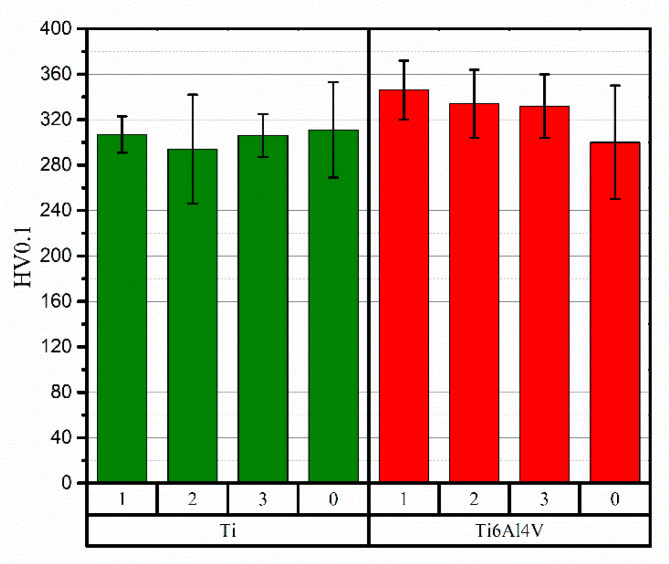
Results of the microhardness measurements.

The average values of Vickers hardness, together with the standard deviations, determined based on 10 indentations made on cross sections are shown in Figure 11. The hardness of the as-deposited Ti coating was 311 ± 42 HV_0.1_. In the case of this material, laser surface treatment did not result in significant changes. The Ti coating not laser-treated, as well as those treated at 1 and 3 ms^−1^, showed very similar average hardness values, while the Ti-2 sample had the lowest hardness of all analysed materials—294 HV_0.1_. For the as-sprayed coatings, the hardness of Ti64 was negligibly lower than Ti (difference c.a. 2%). As a result of the laser beam interaction with the top layers of the coatings, the average hardness values of the Ti64 alloys increased with increasing interaction time up to 346 ± 26 for τ_1_ = 5 × 10^−5^ s. This is an enhancement of about 15% in relation to the hardness of the unmodified coating.

### 3.5. Wetting Angle

**Figure 12:**
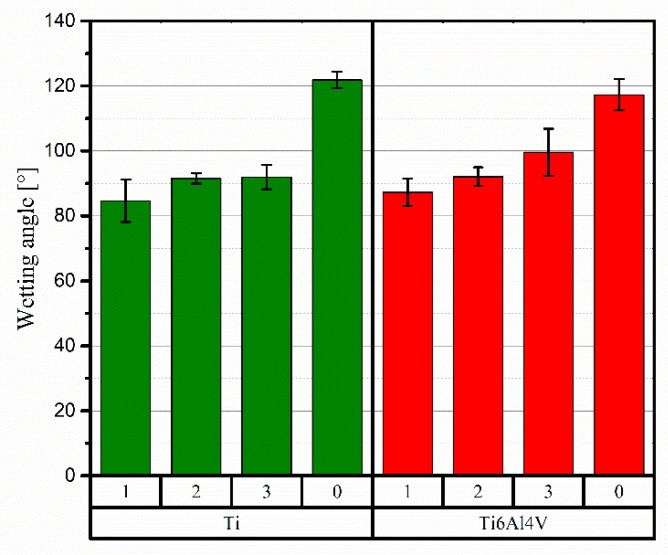
Results of the wetting angle measurements.

The results of the wettability angle measurements, together with corresponding error bars (here as standard deviation), are summarised in Figure 12. For both materials investigated, the highest wettability angle values were measured for the unmodified coatings, i.e., 120° and 118° for Ti and Ti64, respectively. Regardless of the laser beam interaction, τ_i_, time during laser surface treatment, there was a decrease in the wetting angle value, signifying an increase in hydrophilicity, for both Ti and alloy. For both materials, the lowest laser beam velocities led to the greatest decrease in the wettability angle—down to 85° and 87° for Ti and Ti64, respectively. For the Ti64 sample, the decline in this parameter corresponds with decreasing beam interaction time. In the case of pure Ti, there was no significant difference in the value of the wetting angle for surfaces modified with the laser speeds of 2 and 3 ms^−1^.

### 3.6. Surface Roughness

Surface roughness measurements considered the direction of nozzle travel during the CS deposition process. The results of the obtained Ra values in both parallel (‖CS) and perpendicular (ꓕCS) directions are shown in Table 3. In all cases analysed, the Ra values measured in the direction parallel to the CS were significantly lower than the values achieved in the perpendicular direction. For Ti specimens, no significant changes in surface roughness were observed as a result of the employed laser treatment. In contrast, the situation was different for the titanium alloy Ti64. Regardless of the time of interaction, τ_i_, Ra measured in the perpendicular direction decreased with respect to the unlased Ti64 surface. The higher the laser beam scanning speed was, the lower the impact on the Ra value. For v_1_ (1 ms^−1^), Ra reached 25.15 µm, while for v_3_ (3 ms^−1^), Ra was 27.08 µm. Analysing the results in the direction parallel to the CS nozzle for Ti64, the change in surface roughness was only noted for the longest interaction time—from 20.25 µm to 17.48 µm, which is a decrease of approximately 14%.

## 4. Discussion

Results collected from X-ray diffraction phase analysis showed no significant changes caused by the severe plastic deformation occurring during the cold spraying process, or they were below the detection threshold of the method used. Furthermore, using the XRD method, no structural changes in phase composition were observed as a result of the applied laser surface treatment, or, again, they were below the limits of XRD method sensitivity.

The same parameters of the Ti and Ti64 CS processes, shown in Table 1, led to the deposition of the coatings 923 ± 27 and 456 ± 26 µm thick, respectively. In both cases, the interface between the coating and the substrate was defective, but by far a greater number of imperfections was observed for the titanium alloy. Coating thickness measurements showed that the efficiency of the cold spraying processes for the two materials was considerably different. This is explainable on account of the different mechanical and physical properties of Ti and Ti64, the fundamentally distinct morphologies of the feedstock powders, and the distributions of the size particles. The influence of these properties on the ability to form an adhesive bond with the substrate and, thus, the efficiency of the spraying process, was described by Jodoin in the example of an aluminium alloy (Al 2618) sprayed on Al 6061 substrate [38]. Cavaliere underlines that the ability of particles to undergo plastic deformation is a function of the properties of the material and the conditions of the deposition processes [39]. Hussain, in his work, points out that in the case of titanium, in addition to temperature, the powder particle velocity plays a crucial role in the deposition efficiency (DE). One should emphasize that the morphology of the powder can severely influence the particle drag coefficient and, thus, can influence the velocity and acceleration of the said particle [40]. In this case, it is also believed that, indeed, differences in the morphology of the powders were the primary factor behind such extremely different thicknesses of the cold-deposited coatings.

SEM observations of the powders’ morphologies, and subsequent analysis of the CS coatings, have shown that the structure of the powder particles has a considerable influence on multiple aspects. Firstly, it affects the thickness of the deposited layer, secondly, the quality of the bond between the substrate and the coating (both previously mentioned), thirdly, the morphology of the top layer of the coating, and lastly, the way in which this top layer of the coating responds to laser modification by melting. While no significant differences can be seen in the microstructure of the coatings close to the substrate, as both Ti and Ti64 formed a well-dense coating with no visible pores, when observed from the top view, they show great similarity to the structure of the feedstock powders. The increasing density level of cold-sprayed coatings towards the substrate is well known in the literature and is referred to as the tamping effect. It has been described by, e.g., Hussain [36] and Li Cheng et al. [37]. The fact that the top layer of the coating resembles morphologically the feedstock powder indicates that the plastic deformation of the particles was no longer so severe. From the point of view of the subsequent laser processing and its effects, this is a very important conclusion. As can be seen in Figure 6 and Figure 7, the shape of the particles found on the top layer of the coating more or less allows or hinders the spreading of the molten material and its subsequent infiltration of the pores. In the case of titanium, the angular particles, located at the top layers of the coating, impeded the flow of liquid titanium into the voids. The reason may also be that, in this case, the pores had a more irregular size and, therefore, their wettability by the molten titanium was reduced. It is also noteworthy that for the longest interaction time (lowest beam travel speed), the impact of this phenomenon is diminished. Irrespective of the beam interaction time with the Ti6Al4V coating, no such large open pores were observed on the surface as in the case of commercially pure Ti. Hence, the initial powder morphology is indicated as the main reason for this phenomenon occurring during laser surface treatment. Optimisation of the LST conditions in terms of reducing the number of open pores on the surface is of paramount importance. The presence of open pores on the surface can have a significant impact on the properties of the coating, especially its resistance to degradation. The dense coating that exists between the substrate and the surrounding corrosive environment may provide an effective barrier against corrosive phenomena and protect against premature degradation.

The level of residual stress induced in the coating material, e.g., due to the deposition technique used, is of utmost significance for several important reasons. The state of material stress influences, inter alia, fatigue life, wear resistance, corrosion resistance of coated parts, coating’s integrity, and service life [40,41,42,43]. For components working under load, pre-stresses adding up to these stresses result from the loading caused by the external forces and, thereby, reduce or increase the strength (by reducing/increasing the maximum load that can be carried without causing damage or deformation), as well as promote the formation and propagation of cracks from the surface into the material. Based on the stress measurements results shown in Figure 10, it can be concluded that the values for both materials, Ti and Ti64, subjected to laser treatment, are very similar. The results obtained exhibit very similar correlations relating stress level and laser beam interaction time. In all cases, these stresses are tensile and increase with decreasing laser beam speed, *v_i_*. For samples Ti-1 and Ti64-1, these stresses reach significantly high values of over 200 MPa and must not be neglected when designing and predicting the optimum operational conditions for CS elements after LST. The stresses arising from the interaction between the bulk material (or material’s particles) and the laser beam have been extensively analysed and described in the literature [44,45], as well as for a deeper insight into the processes involved in SLM 3D printing. Simson et el. [46] described two basic stress-inducing mechanisms: (a) the temperature gradient mechanism, and (b) the cool-down mechanism. Although their work focused on stresses induced during SLM processes, the mechanisms they proposed are also analogous and might be applied to laser surface treatment. The introduction of residual stresses into the material results from the increasing interaction time. It is connected mostly with the thermal expansion of material. Relatively small areas after melting are cooled down with the high rates. This causes material shrinkage, which affects the nearby area tensions. The compressive stresses are introduced mostly by a huge difference of coefficients of thermal expansion (substrate material: ~22 × 10^−6^ K^−1^, Ti and Ti64: ~9 × 10^−6^ K^−1^). Longer interaction time increases the amount of energy delivered to the material, raising the temperature difference. The higher temperature of Al-base results in higher expansion. After cooling down, this introduces compressive stress to the layer material.

An intriguing situation that differs from those described earlier by Fardan [43], Hussain [40], and Price et al. [47] occurs for residual stresses in the material after the cold spray deposition process. While for Ti coatings the presence of compressive stresses is in correlation with the aforementioned studies and results from the kinetic impact of sprayed particles and their plastic deformation, the presence of tensile stresses for Ti64 is less common in the literature. According to Suhonen et al., tensile stresses in sprayed coatings indicate the domination of the quenching effect over the peening effect [48]. For the sake of the coating’s performance, compressive stresses are preferred. Therefore, further optimisation of spraying parameters is suggested for Ti64 coatings.

Based on the results of the Vickers hardness measurements, it can be concluded that, for the Ti coating, conducted laser treatment had no significant effect on the HV_0.1_. Although the slightly lower average value for sample Ti-2 was observed, the high standard deviation values for this sample and Ti-0, may lead to the conclusion that de facto hardness has not changed. The contrary was observed for coating samples deposited from titanium alloy. In this case, samples treated at 2 and 3 ms^−1^ showed similar microhardness, which was over 10% higher than as-deposited coating. For the longest interaction time, an increase of 15% HV_0.1_ was recorded. Several reasons for the hardness increase after laser processing are found in the literature. Researchers attribute the cause of the elevated hardness to an increase in stress levels, phase transformations resulting in the formation of fine or ultrafine precipitates, or enhanced dislocation density [49,50]. It is worth emphasizing that the results of the hardness measurements showed a large scatter of values. Similar observations for cold-sprayed coatings were had by Maharajan et al. [49] (which they also tested using the Vickers method at the same loading). According to Hussain, the main challenge during hardness measurements of CS coatings is associated with the presence of porosity. It is the source of large scatterings of values and, therefore, definitive conclusions should be drawn very carefully. It is reasonable that this effect can mainly be attributed to the microstructure features of cold-sprayed materials but is also combined with the method limitations themselves.

Wetting angle measurements of CS titanium-based materials, and analyses of LST-induced changes, are important from the point of view of potential implementation in biomedical applications [51]. The wettability of the materials was determined in the same conditions and using the exact same liquid (deionised water), so the influence of the surface tension of the liquid (therefore its purity and temperature) may be disregarded as the main factor causing alterations. Thus, the most likely cause is a change in surface free energy resulting from the applied LST that modified the material’s state. The Ra results can be disregarded (the effect of surface roughness), because, as was apparent from the examples analysed, there was no change in Ra for Ti, and for Ti64 it was slight; however, the wetting angle varied in both cases. Clearly, the influence on the energy state was the largest for the longest interaction times of the laser beam with the coating material.

The results of surface roughness measurements, represented by the Ra parameter, are listed in the Table 3. Due to the characteristic of the cold spray process, which is the deposition of powder from a nozzle travelling in straight lines (which is intended to achieve a uniform coating thickness over the entire target substrate surface), it was decided to measure Ra in two directions orthogonal to each other. Based on the results obtained, it can be concluded that, in the case of Ti coatings, the LST did not result in significant Ra changes. These remained almost constant relative to the values obtained for the as-deposited coating. The highest difference in the Ra value of the surface was for laser processing of Ti64 alloy performed with the lowest beam scanning speed. In both directions, perpendicular and parallel to the CS nozzle, its value dropped by 14%. A previous analysis of the SEM images of Ti coatings, taken before and after LST, led to the conclusion that the morphology of the powder was of high significance. The angular particles impeded the flow of molten Ti and closed the open pores. It also led to the preservation of the surface roughness state after contact with the laser; whereas, the reduced Ra value for coatings with Ti6Al4V may be attributed to the facilitated filling of open pores stemming from superior wettability (in comparison to angular Ti particles).

## 5. Conclusions

This work presents results of a study on the effect of laser beam interaction time used for surface modification on the properties of Ti and Ti6Al4V coatings cold-sprayed on an Al7075 aluminium alloy substrate. Among the most noteworthy conclusions, the substantial role of the morphology of the feedstock powder on the response of the top layers of the coating material to laser treatment was identified. Preserved, almost non-deformed particle shapes may prevent the free flow of laser-melted material and thereby hinder filling open pores. The analyses performed determined the influence of laser treatment on the residual stress state in the cold-sprayed coating. The longer interaction time of the laser beam with the topmost layer of the coating led to higher residual stresses. For titanium alloy, a 14% increase in Vickers microhardness was observed, compared to the as-sprayed material. The surfaces of both materials, Ti and Ti64, regardless of the laser processing parameters applied, showed increased hydrophilicity, the cause of which has been attributed to changes in the energy state of the surface.

## Figures and Tables

**Table 1:** Parameters of the cold-spray processes.

Parameter	Value
Temperature [°C]	775
Pressure [MPa]	4
Propellant gas	50% N_2_ + 50% He
Nozzle travel speed [mm∙s^−1^]	300
Powder feeding rate [g∙min^−1^]	90
Step size [mm]	2
Standoff distance [mm]	40
Number of layers	3

**Table 2:** Parameters of the laser post-processing treatment.

Sample		Laser Power, *P* [W]	Spot Diameter, *d* [µm]	Power Density [W∙mm^−2^]	Protective Gas	Scanning Speed, *v_i_* [m∙s^−1^]	Interaction Time, *τ_i_* [s]	Overlapping [%]
Ti-0;	Ti64-0	-	-	-	-	-	-	-
Ti-1;	Ti64-1	150	50	7.6 × 10^4^	Ar	1	5.0 × 10^−5^	50
Ti-2;	Ti64-2	150	50	7.6 × 10^4^	Ar	2	2.5 × 10^−5^	50
Ti-3;	Ti64-3	150	50	7.6 × 10^4^	Ar	3	1.7 × 10^−5^	50

**Table 3:** Surface roughness measurement results.

Sample	Ra [µm]	
	ꓕCS	‖CS
Ti-0	20.44 ± 1.14	16.87 ± 1.22
Ti-1	20.14 ± 0.14	15.31 ± 1.93
Ti-2	21.23 ± 2.84	15.22 ± 1.85
Ti-3	20.84 ± 0.60	16.85 ± 0.88
Ti64-0	29.10 ± 0.44	20.25 ± 2.25
Ti64-1	25.15 ± 1.00	17.48 ± 1.83
Ti64-2	25.31 ± 1.84	20.09 ± 2.53
Ti64-3	27.08 ± 2.37	20.87 ± 1.02
